# General Control of Amino Acid Synthesis 5-Like 1-Mediated Acetylation of Manganese Superoxide Dismutase Regulates Oxidative Stress in Diabetic Kidney Disease

**DOI:** 10.1155/2021/6691226

**Published:** 2021-02-17

**Authors:** Tingting Lv, Yao Lu, Yi Liu, Hong Feng, Chensheng Li, Wei Sheng, Zhengguo Cui, Suwei Zhu, Xia Gu, Zhe Yang, Qiang Wan

**Affiliations:** ^1^School of Medicine, Cheeloo College of Medicine, Shandong University, Jinan, Shandong 250012, China; ^2^Department of Pulmonary and Critical Care Medicine, Shandong Provincial Hospital, Cheeloo College of Medicine, Shandong University, Jinan, Shandong 250012, China; ^3^Cancer Centre, Shandong Provincial Hospital, Cheeloo College of Medicine, Shandong University, Jinan, Shandong 250012, China; ^4^Department of Gastrointestinal Surgery, Shandong Provincial Hospital, Cheeloo College of Medicine, Shandong University, Jinan, Shandong 250012, China; ^5^Department of Public Health, Graduate School of Medicine and Pharmaceutical Sciences, University of Toyama, 2630 Sugitani, Toyama 930-0194, Japan; ^6^Department of Endocrinology, Shandong Provincial Hospital, Cheeloo College of Medicine, Shandong University, Jinan, Shandong 250012, China

## Abstract

Diabetic kidney disease (DKD) is the major cause of end-stage renal disease (ESRD). In the past few decades, there has been a large amount of evidence to highlight the pivotal role of oxidative stress in the development and progression of DKD. However, the detailed molecular mechanisms are not fully elucidated. A new sight has been established that the mitochondrial acetyltransferase GCN5L1 participates in cellular redox homeostasis maintenance in DKD. Firstly, we found that the expression of GCN5L1 is significantly elevated both in human and mouse kidney tissues with DKD and in hyperglycemic renal tubular epithelial cells (TECs), while deletion of GCN5L1 could effectively ameliorate oxidative stress-induced renal injury in DKD. Furthermore, deletion of GCN5L1 could reduce MnSOD acetylation on lysine 68 and activate its activity, thereby scavenging excessive ROS and relieving oxidative stress-induced renal inflammation and fibrosis. In general, GCN5L1-mediated acetylation of MnSOD exacerbated oxidative stress-induced renal injury, suggesting that GCN5L1 might be a potential intervention target in DKD.

## 1. Introduction

Diabetic kidney disease (DKD) is one of the most common microvascular complications of diabetes mellitus and has become the leading cause of end-stage renal disease (ESRD), a worldwide public concern [1, 2]. The current clinical interventions for DKD are mainly to control risk factors such as hyperglycemia, hypertension, and proteinuria, which focus on relieving symptoms and delaying the progression of DKD, but their efficacy is limited [3–5]. Therefore, there is a strong clinical need to further explore the pathophysiological mechanism of DKD and find potential intervention targets.

Recently, hyperglycemia-induced oxidative stress is increasingly being seen as a major pathogenic mechanism that leads to kidney damage in DKD, which is caused by the overproduction of reactive oxygen species (ROS) induced by disturbance of mitochondrial respiration [6]. As a star molecule of antioxidant enzymes, manganese superoxide dismutase (MnSOD) could effectively scavenge the toxic ROS by catalyzing the dismutation of superoxide anion radical (O_2_^·-^) to hydrogen peroxide (H_2_O_2_) and molecular oxygen (O_2_) [7, 8]. The conventional perspective is that the ability of MnSOD to scavenge ROS depends on its abundance, while a novel view believes that it depends more on the enzymatic activity [9]. It has been reported that the reversible lysine acetylation of MnSOD could regulate its detoxification activity [10]. However, the role of acetylated MnSOD in DKD and its upstream regulatory mechanisms have been largely unaddressed in the past.

General control of amino acid synthesis 5-like 1 (GCN5L1) is a novel molecule with sequence homology to the nuclear acetyltransferase GCN5. It predominantly locates in the mitochondria to promote mitochondrial protein acetylation and interacts with canonical substrates of SIRT3 to counter its activity [11]. Current studies indicate that GCN5L1 is involved in regulating multiple mitochondrial biological functions such as mitophagy, mitochondrial biogenesis, and fatty acid oxidation (FAO) [12–14]. In the present study, we supplemented the important role of GCN5L1 in the maintenance of cellular redox homeostasis in DKD. We found that the expression of GCN5L1 is significantly elevated both in vivo in kidney tissues from DKD patients and mouse models and in vitro renal tubular epithelial cells (TECs) treated with high glucose, while reducing GCN5L1 expression could effectively attenuate mitochondrial oxidative stress, epithelial-to-mesenchymal transition (EMT), and inflammation induced by high glucose. Furthermore, downregulated GCN5L1 in TECs stimulated with high glucose activates MnSOD by decreasing the acetylation level of its K68 site and alleviates hyperglycemia-induced renal damage. These findings shed new light on the pathophysiological mechanism of DKD and hint GCN5L1 might be a potential intervention target.

## 2. Materials and Methods

### 2.1. Cell Lines

Human renal proximal tubular epithelial cells (HK-2) were purchased from the American Type Culture Collection (ATCC) and sterilely cultured with 10% FBS and 1% penicillin/streptomycin in DMEM at 37°C and 5% CO_2_.

### 2.2. Human Renal Biopsy Samples

Renal biopsy specimens from type 1 diabetes patients (*n* = 6) with pathological diagnosis of DKD were obtained from Department of Pathology, Qilu Hospital of Shandong University. The control samples (*n* = 6) were taken from healthy kidney poles of individuals who underwent cancer nephrectomy without other renal diseases. All procedures were approved by the Ethics Review Committee of Shandong University (ECSBMSSDU2018-1-045) and performed in accordance with the principles of the Helsinki Declaration after obtaining informed consent from the patients.

### 2.3. Animals

Male C57BL/6 mice (6-8 weeks old) were purchased from Shandong University Experimental Animal Centre. All animal experiments were performed according to the protocols approved by Animal Ethics Committee of Shandong University. The mice were randomly divided into the following experimental groups: normal control mice (non-DN group, *n* = 6), streptozotocin-induced DN mice (DN group, *n* = 9), AAV-empty vector DN mice (AAV-vector DN group, *n* = 9), and AAV-GCN5L1 DN mice (AAV-GCN5L1 DN group, *n* = 9). For adenovirus-associated virus experiments, mice were anaesthetized firstly and then were injected with AAV2/9-HU6-shGCN5L1 (titer: 1 × 10^12^ vg/ml) or AAV2/9-HU6-Scramble (titer: 1 × 10^12^ vg/ml) into renal cortex in situ. The target sequence of shGCN5L1 was 5′-GAAGAGGAGGAGAGAAGCTAT-3′. The sequence of negative control was 5′-TTCTCCGAACGTGTCACGT-3′. After a week recovery, the DN groups were given an intraperitoneal injection of streptozotocin (50 mg/kg) after 12 hours fasting for five consecutive days. The control group was treated with an equal volume of citric acid buffer solution. One week after STZ injection, mice with random blood glucose levels over 16.7 mM were considered diabetes [15–17]. Mice with random blood glucose levels below 16.7 mM were excluded from the experiment; the DN group excluded three mice (*n* = 6), the AAV-vector DN group excluded one mouse (*n* = 8), and the AAV-GCN5L1 DN group excluded one mouse (*n* = 8). The actual blood glucose data of mice is shown in Supplementary Table S1. Then, all mice were housed in temperature-controlled rooms with a 12-hour light-dark cycle and had free access to food and drinking water for sixteen weeks.

### 2.4. Histopathology and Immunohistochemistry

Kidney tissues were fixed in 4% buffered formalin and embedded in paraffin. Sections were then stained with hematoxylin-eosin (HE), periodic acid-Schiff (PAS), and Masson's trichrome. For immunohistochemistry staining, sections were retrieval antigens in sodium citrate buffer after deparaffinized and rehydrated and then incubated with the antibody at 4°C overnight. Afterwards, the kidney sections were incubated with HRP-conjugated secondary antibody for 1 h shaking at room temperature. Diaminobenzidine (DAB) was applied to visualize the signal. Images were captured by a Nikon light microscope. Image analysis was performed using ImageJ software.

### 2.5. Renal Function Measurements

Mice were placed in metabolic cages for 24 hours to collect urine samples. Blood samples were collected by cardiac puncture after mice was anesthetized at the end of the experiment. Serum creatinine and urinary protein were measured by using commercial determination kits (Nanjing Jiancheng Bioengineering Institute, China) according to the manufacturer's instructions.

### 2.6. Western Blotting Analysis

Proteins of cells or kidney tissues were extracted by using RIPA buffer supplemented with 1% protease inhibitor and phosphatase inhibitors. After diluted in 4× SDS-PAGE loading buffer and denatured in 95°C for 10 min, samples were separated by SDS-PAGE and subsequently transferred to PVDF membranes. The membranes were blocked with 5% skimmed milk and then incubated with rabbit anti-GCN5L1 antibody (Proteintech, 19687-1-AP), mouse anti-GCN5L1 antibody (Santa Cruz, sc515444), rabbit anti-SOD2/MnSOD (acetyl K68) antibody (Abcam, ab137037), rabbit anti-MnSOD antibody (Proteintech, 24127-1-AP), rabbit anti-NLRP3 antibody (Abcam, ab210491), mouse anti-caspase-1 antibody (Santa Cruz, sc392736), rabbit anti-IL18 antibody (Proteintech, 10663-1-AP), rabbit anti-IL1beta antibody (ABclonal, A11370), rabbit anti-E-cadherin antibody (Proteintech, 20874-1-AP), and rabbit anti-*α*SMA antibody (Proteintech, 55135-1-AP). Then, the membranes were incubated with the corresponding secondary antibody (HRP-tagged goat anti-mouse or anti-rabbit IgG) and detected by enhanced chemiluminescence reagents (ECL, Millipore, USA). Quantitative analysis was performed using the ImageJ software.

### 2.7. Immunofluorescence Staining

Cells were fixed with 4% paraformaldehyde for 20 minutes, permeabilized in 0.5% Triton X-100 for 10 minutes, and then blocked with 1% goat serum for 1 hour at room temperature. Then, cells were incubated with mouse anti-GCN5L1 antibody (Santa Cruz, sc515444), rabbit anti-E-cadherin antibody (Proteintech, 20874-1-AP), and rabbit anti-*α*SMA antibody (Proteintech, 55135-1-AP) overnight at 4°C. Secondary antibody (Alexa Fluor 488 Goat Anti-Rabbit IgG H&L, ab150077, 1 : 500) was used to stain the cells, and DAPI nuclear stain was used to counterstain. Images were obtained using a Nikon microscope.

### 2.8. Intercellular ROS

In vitro, intercellular ROS was determined by the addition of MitoSOX (5 *μ*M) to the cells which incubated at 37°C for 10 min in darkness. In vivo, superoxide production in renal tissues was measured in frozen kidney sections exposed to 6 *μ*m dihydroethidium (DHE) at 37°C for 30 min and protected from light (DHE, Beyotime Institute of Biotechnology, Shanghai, China). The fluorescence intensity of intracellular ROS was captured using Nikon microscope imaging system (Nikon, Tokyo, Japan).

### 2.9. MnSOD Activity

The MnSOD activity was analysed in the kidney cortex and cell homogenates using commercial kits according to the manufacturer's instructions (WST-8; Beyotime Institute of Biotechnology, Shanghai, China).

### 2.10. Immunoprecipitation

The immunoprecipitation assay was performed as described previously [18]. In brief, 1 mg cell lysates was incubated with 10 *μ*g antibody and 10 *μ*l protein A/G resin at 4°C overnight. Next day, the protein was eluted from the resin with elution buffer and analysed by western blotting assay.

### 2.11. In Situ Proximity Ligation Assay (In Situ PLA)

The in situ proximity ligation assay was carried out as described previously to detect the interaction between GCN5L1 with MnSOD using mouse anti-GCN5L1 antibody (Santa Cruz, sc515444) and anti-MnSOD antibody (Proteintech, 24127-1-AP) [19]. Representative images of the fluorescence were obtained using a Nikon microscope.

### 2.12. Quantitative Real-Time PCR

Total RNA was extracted from specimens using TRIzol reagent and converted to cDNA using reverse transcriptase kits (Takara, RR047A). qRT-PCR was carried out using SYBR Premix Ex Taq II (Takara, RR820L) as described previously [19]. Sequence-specific primers used were as follows: mouse-GCN5L1 Fwd: 5′-AGAACTGGGCTAGGAGCATC-3′, Rev: 5′-AGCTGCCCTTTGTAGACGTA-3′ and mouse-*β*-actin Fwd: 5′-TGCGTGACATCAAAGAGAAG-3′, Rev: 5′-TCCATACCCAAGAAGGAAGG-3′.

### 2.13. Statistical Analysis

Each experiment was performed at least for three times. All data were analysed using GraphPad Prism 6.0, with Student's *t*-test (comparison between two groups) or one-way ANOVA (comparison among multiple groups). *P* < 0.05 was considered to be statistically significant.

## 3. Results

### 3.1. The Expression of GCN5L1 Is Significantly Elevated in Kidney Tissues from Diabetic Kidney Disease Patients and Mouse Models

**Figure 1:**
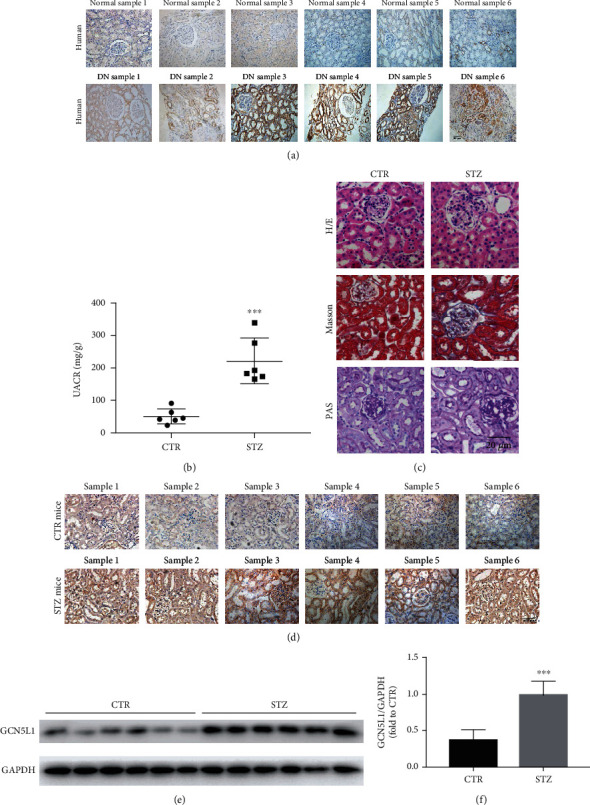
The expression of GCN5L1 is significantly elevated in kidney tissues from diabetic kidney disease patients and mouse models. (a) Immunohistochemistry staining for GCN5L1 expression in human renal biopsies. Scale bars = 50 *μ*m. (b) Urine albumin-to-creatine ratio (UACR) in STZ-induced diabetic mice. Independent experiments were performed in triplicate. (c) Hematoxylin and eosin staining, Masson's trichrome staining, and periodic acid-Schiff staining in the kidneys from STZ-induced diabetic mice. Scale bars = 20 *μ*m. (d) Immunohistochemistry staining for GCN5L1 expression in mouse kidney tissues. Scale bars = 50 *μ*m. (e, f) Western blotting analysis of GCN5L1 protein expression in the kidneys from STZ-induced diabetic mice. Data are presented as the mean ± SD. *n* = 6; ^∗∗∗^*P* < 0.001.

To investigate whether GCN5L1 expression is associated with diabetic kidney disease, IHC staining was performed to evaluate the protein levels of GCN5L1 in renal tissues. Normal tissues from adjacent normal tissues of patients with renal cell carcinoma and kidney biopsy samples from patients with diabetic kidney disease were used in this study. The results showed that the protein level of GCN5L1 underwent significant increase in the human kidneys with DKD, which was measured by IHC (Figure 1(a)). To further study the role of GCN5L1 in DKD, we next established a DKD mouse model by STZ treatment. The renal injury of STZ-induced DKD mice was confirmed by enhanced urine albumin-to-creatinine ratio (UACR) (Figure 1(b)). Renal morphological examination by hematoxylin and eosin staining, Masson's trichrome staining, and periodic acid-Schiff staining indicated that mesangial matrix deposition and renal tubule vacuolation were aggravated in STZ-induced DN mice compared with control mice (Figure 1(c)). The GCN5L1 expression was also increased in kidney tissues of STZ-induced DKD mice by IHC and western blotting (Figures 1(d)–1(f)). These results showed that GCN5L1 was overexpressed in the DKD kidneys, which indicated the possible involvement of GCN5L1 in the pathogenesis and progression of DKD.

### 3.2. Reduction of GCN5L1 Ameliorates Renal Tubulointerstitial Injury and Proteinuria in DKD Mice

**Figure 2:**
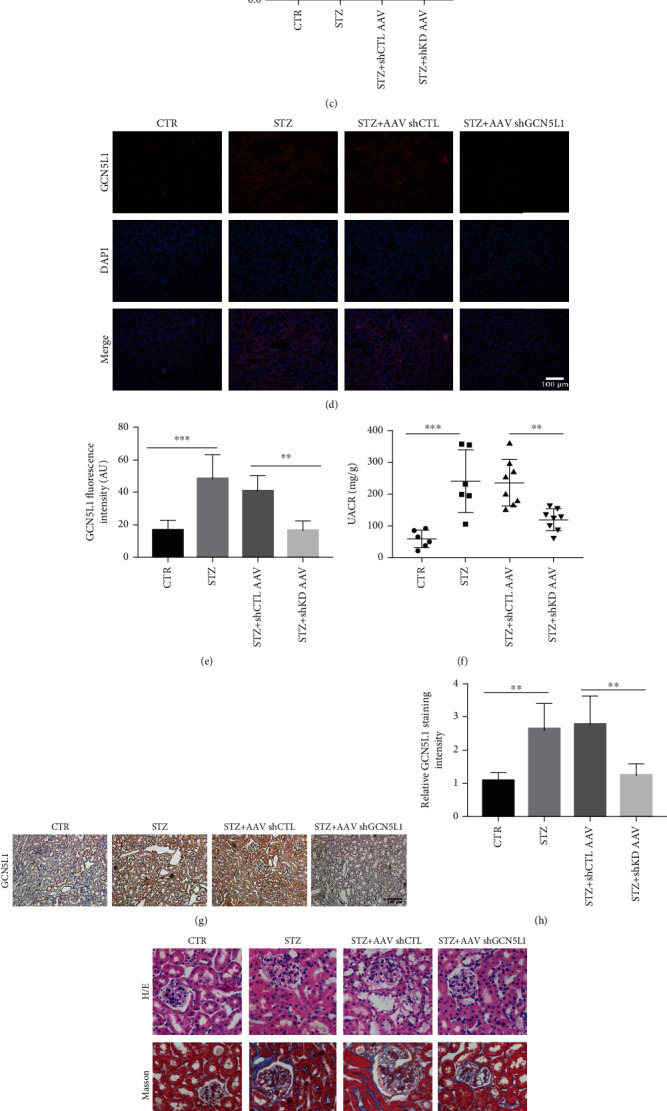
Reduction of GCN5L1 ameliorates renal tubulointerstitial injury and proteinuria in DKD mice. (a) Experimental design for STZ-induced diabetic GCN5L1 knockdown mice. (b, c) Western blotting analysis showing the expression of GCN5L1 in the kidneys from diabetic GCN5L1 knockdown mice. Data are presented as the mean ± SD. (d, e) Immunofluorescent staining and fluorescence intensity for GCN5L1 expression in kidney sections. Scale bars = 100 *μ*m. (f) Urine albumin-to-creatinine ratio (UACR) in the kidneys from diabetic GCN5L1 knockdown mice. Independent experiments were performed in triplicate. (g, h) Immunohistochemistry staining and relative staining intensity for GCN5L1 expression in kidney sections. Scale bars = 100 *μ*m. (i) Hematoxylin and eosin staining, Masson's trichrome staining, and periodic acid-Schiff staining in the kidneys from diabetic GCN5L1 knockdown mice. Scale bars = 20 *μ*m. *n* = 6‐8; ^∗∗^*P* < 0.01 and ^∗∗∗^*P* < 0.001.

To further elucidate the importance of GCN5L1 in regulating renal injury, GCN5L1 shRNA-expressing adenovirus-associated virus was used to induce kidney-specific GCN5L1 knockdown (KD) mice. Firstly, GCN5L1 shRNA-expressing AAV was locally injected into the renal cortex of C57BL/6 mice, along with STZ treatment for about sixteen weeks to generate diabetic GCN5L1 KD mice (Figure 2(a)). The efficiency of GCN5L1 knockdown in the kidney was confirmed by western blotting, immunofluorescence staining, and IHC staining (Figures 2(b)–2(e), 2(g), and 2(h)). Then, we detected urinary albumin and creatinine to explore the effect of GCN5L1 in diabetic-induced renal injury. As shown in Figure 2(f), the levels of urine albumin-to-creatinine ratio (UACR) were reduced in diabetic GCN5L1 KD mice compared with the control AAV-treated group, indicating that GCN5L1 KD alleviated STZ-induced renal injury. Consistently, in renal morphological analysis, diabetic GCN5L1 KD mice displayed decreased mesangial matrix expansion and renal tubule vacuolation in renal tissues compared with the diabetic control AAV group (Figure 2(i)). Together, these observations suggested that GCN5L1 plays a critical role in diabetic renal damage.

### 3.3. Downregulation of GCN5L1 Activates MnSOD to Scavenge ROS by Reducing MnSOD Acetylation on Lysine 68 in TECs Treated with High Glucose

**Figure 3:**
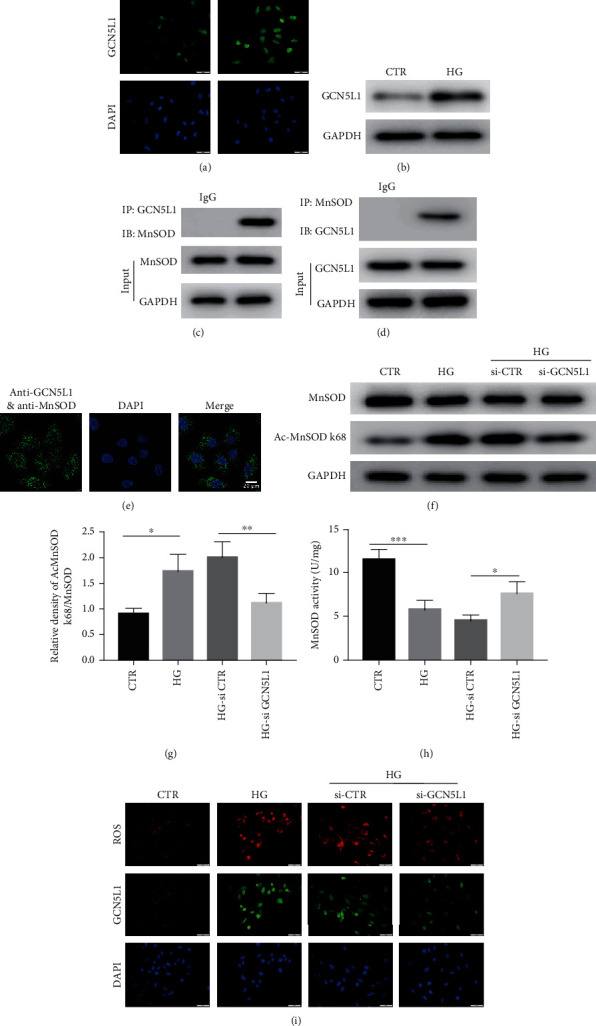
Downregulation of GCN5L1 activates MnSOD to scavenge ROS by reducing MnSOD acetylation on lysine 68 in TECs treated with high glucose. (a, b) Immunofluorescent staining and western blotting analysis of GCN5L1 protein expression in TECs exposed to high glucose. Scale bars = 50 *μ*m. (c, d) Coimmunoprecipitation of MnSOD and GCN5L1 in TECs. (e) Interaction of MnSOD and GCN5L1 in TECs visualized by Duolink proximity ligation assay. (f, g) Western blotting analysis of the protein levels of MnSOD and Ac-MnSOD K68 in TECs exposed to high glucose following GCN5L1 knockdown. (h) MnSOD activity was assessed in TECs exposed to high glucose following GCN5L1 knockdown. Independent experiments were performed in triplicate. (i) Cellular ROS was detected by MitoSOX staining in GCN5L1 knockdown TECs treated with high glucose. Scale bars = 50 *μ*m. Independent experiments were performed in triplicate. ^∗^*P* < 0.05, ^∗∗^*P* < 0.01, and ^∗∗∗^*P* < 0.001.

In order to explore the specific mechanism of GCN5L1-mediated renal injury in DKD, we first applied tubular epithelial cells (TECs) to test the expression of GCN5L1 under high glucose in vitro. As shown in Figures 3(a) and 3(b), immunofluorescence staining and western blotting analysis revealed a significant elevation of GCN5L1 in TECs treated with high glucose. MnSOD, a major mitochondrial antioxidant enzyme, located in mitochondria, scavenged cellular ROS and was proved to exert an antioxidative stress effect in hyperglycemia-induced renal injury. Moreover, several studies have demonstrated that MnSOD is modified by acetylation and the acetylation level of MnSOD affects its enzyme activity and its ability of removing cellular ROS [10]. Mitochondria producing excessive ROS lead to oxidative stress in various energy excess conditions, including hyperglycemia [20]. We hypothesized that GCN5L1 may modulate the enzyme activity of MnSOD by acetylation. First, we evidenced that MnSOD physically interacts with GCN5L1 by coimmunoprecipitation assay (Co-IP) and in situ proximity ligation assay (in situ PLA) (Figures 3(c)–3(e)). Then, the data showed that the acetylation level of MnSOD lysine 68 was upregulated after high-glucose treatment, while knockdown GCN5L1 could decrease the acetylation level of MnSOD^K68^ in high-glucose conditions (Figures 3(f) and 3(g)). Finally, to explore the further effect of GCN5L1 on MnSOD enzymatic detoxification activity, the MnSOD enzymatic activity and MitoSOX staining were determined. We found that GCN5L1 deletion rescued the enzymatic activity of MnSOD and diminished cellular ROS production under high-glucose conditions (Figures 3(h) and 3(i)).

### 3.4. Suppression of GCN5L1 Alleviates Inflammation and EMT through the MnSOD/ROS Pathway in TECs under High Glucose

**Figure 4:**
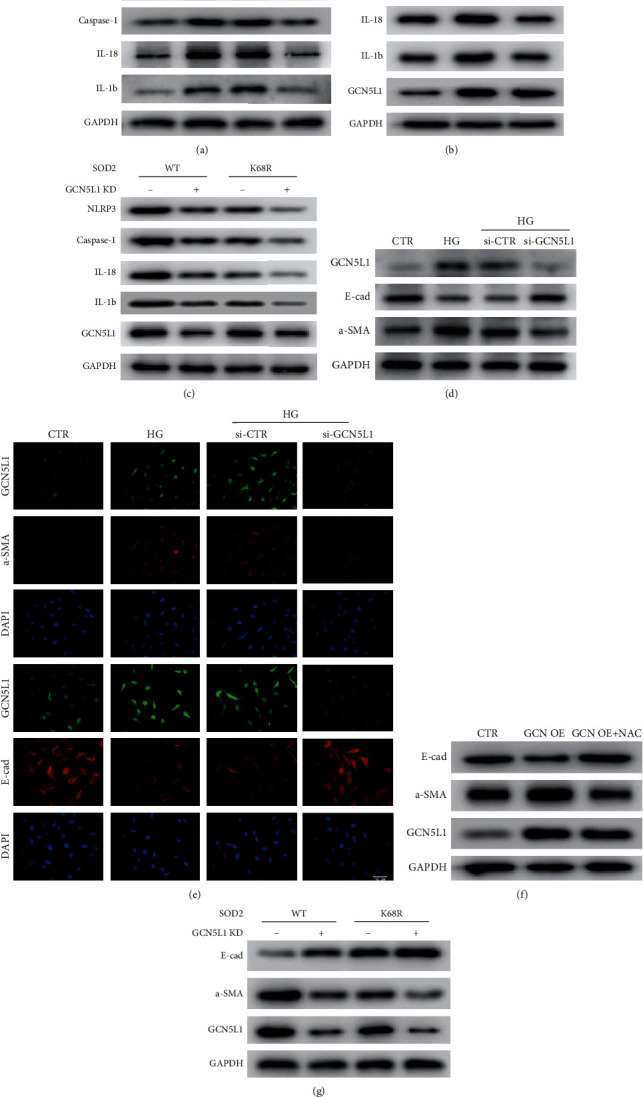
Suppression of GCN5L1 alleviates inflammation and EMT through the MnSOD/ROS pathway in TECs under high glucose. (a) Protein levels of NLRP3, caspase-1, IL18, and IL1beta were detected by western blotting in TECs exposed to high glucose following knockdown of GCN5L1. (b) Protein levels of NLRP3, caspase-1, IL18, and IL1beta were detected by western blotting in GCN5L1 overexpressed TECs treated with NAC. (c) Western blotting analysis of the protein levels of NLRP3, caspase-1, IL18, and IL1beta after cotransfected with MnSODK68-R mutant plasmid and GCN5L1 silencing siRNA. (d, e) Western blotting analysis and immunofluorescent staining for E-cadherin and *α*-SMA in TECs exposed to high glucose following knockdown of GCN5L1. Scale bars = 100 *μ*m. (f) Protein levels of E-cadherin and *α*-SMA were detected by western blotting in GCN5L1 overexpressed TECs treated with NAC. (g) Western blotting analysis of the protein levels of E-cadherin and *α*-SMA after cotransfected with MnSODK68-R mutant plasmid and GCN5L1 silencing siRNA. Independent experiments were performed in triplicate.

Aberrant mitochondrial ROS production caused by diabetes is critical for NLRP3 inflammasome activation via the oxidation of mitochondrial DNA [21, 22]. Western blotting analysis revealed that the expression of NLRP3, caspase 1, IL18, and IL1b was enhanced in TECs treated with high glucose. In contrast, these protein expressions were completely reversed by GCN5L1 knockdown (Figure 4(a)). This suggested that GCN5L1 deletion may inhibit inflammation by eliminating cellular ROS production. To prove this hypothesis, N-acetyl-L-cysteine (NAC), a ROS inhibitor, was employed in GCN5L1-overexpressed TECs. Notably, NAC effectively blocked the GCN5L1 overexpression-mediated upregulation of NLRP3, caspase 1, IL18, and IL1b (Figure 4(b)). Then, transfection of the MnSOD^K68-R^ mutant (lysine residue was replaced by arginine to mimic the deacetylated state) plasmid into GCN5L1 knockdown TECs significantly decreased the expression of NLRP3, caspase 1, IL18, and IL1b (Figure 4(c)). The results revealed that GCN5L1 acetylates MnSOD^K68-R^ to affect inflammation through aberrant ROS-induced oxidative stress.

**Figure 5:**
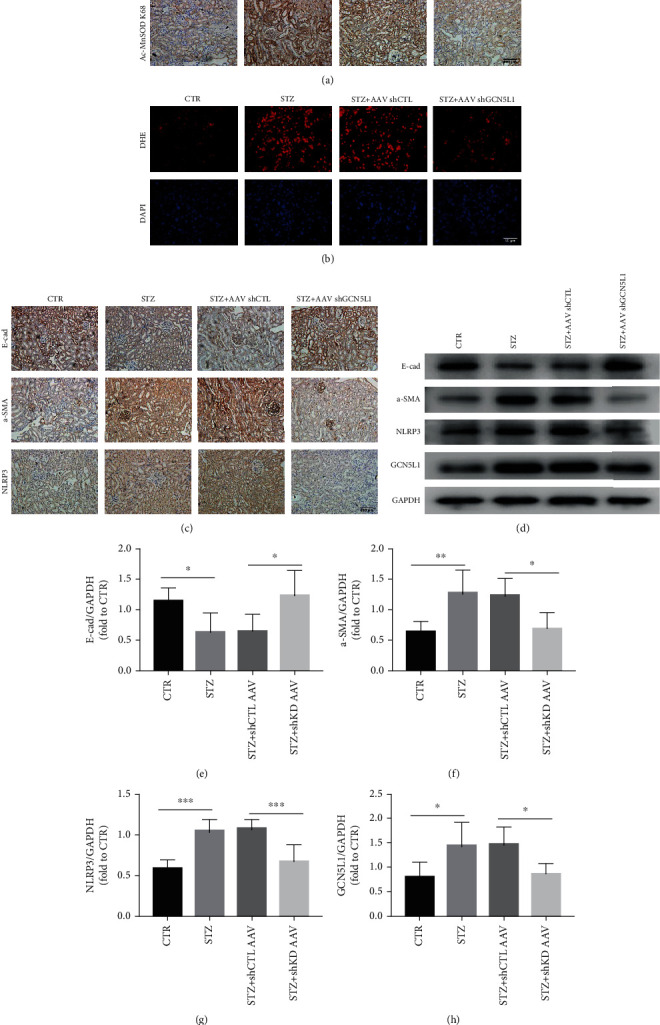
Downregulation of GCN5L1 protects the kidney from hyperglycemia-induced EMT and inflammation via the MnSOD/ROS pathway in vivo. (a) Immunohistochemistry staining for acetylation level of MnSODK68 in the kidneys from diabetic GCN5L1 knockdown mice. Scale bars = 100 *μ*m. (b) DHE fluorescence to detect ROS in the kidneys from diabetic GCN5L1 knockdown mice. Scale bars = 50 *μ*m. (c) Immunohistochemistry staining for E-cadherin, *α*-SMA, and NLRP3 expression in the kidneys of mice. Scale bars = 100 *μ*m. (d–h) Western blotting analysis showing the expressions of E-cad, *α*-SMA, and NLRP3 in the kidneys of mice. Data are presented as the mean ± SD. *n* = 6‐8; ^∗^*P* < 0.05, ^∗∗^*P* < 0.01, and ^∗∗∗^*P* < 0.001.

EMT could also be activated via reactive oxygen species and contributes to renal fibrosis related to diabetic nephropathy [23]. Under this context, whether GCN5L1 has an effect on EMT through the MnSOD/ROS pathway under high-glucose conditions has also aroused our attention. First, the epithelial marker E-cadherin and mesenchymal marker *α*-SMA were evaluated by both western blotting and immunofluorescence staining. As shown in Figures 5(d) and 5(e), E-cadherin was significantly decreased and *α*-SMA was obviously enhanced in TECs under high glucose, while knockdown of GCN5L1 could effectively reverse the expression of E-cadherin and *α*-SMA. In addition, the effects of ROS inhibitor NAC reversed the expression of E-cadherin and *α*-SMA mediated by GCN5L1 overexpression (Figure 4(f)). Then, we detected whether the effect of GCN5L1 on EMT was mediated by MnSOD acetylation. The roles of it were determined in TECs by cotransfection of siRNA of GCN5L1 and MnSOD^K68-R^ mutant plasmid. Western blotting showed that GCN5L1 knockdown resulted in an increased expression of E-cadherin and a decreased expression of *α*-SMA, which was significantly reversed by additional MnSOD^K68-R^ mutants (Figure 4(g)). All the results above suggested that GCN5L1 could acetylate MnSOD^K68^, thereby mediating inflammation and EMT through mitochondrial ROS in vitro.

### 3.5. Downregulation of GCN5L1 Protects the Kidney from Hyperglycemia-Induced EMT and Inflammation via the MnSOD/ROS Pathway In Vivo

Then, we further explored the effect and mechanism of GCN5L1 on diabetic kidney injury in vivo. First, we determined the acetylation level of MnSOD^K68^ in the mouse kidneys. Immunohistochemistry staining showed that GCN5L1 KD greatly diminished acetylation level of MnSOD^K68^ induced by STZ (Figure 5(a)). Then, as stained with ROS-sensitive vital dye DHE, we found the enhanced ROS production induced by STZ in the mouse kidneys was abolished by silencing GCN5L1 (Figure 5(b)). Finally, immunohistochemistry assay revealed that diabetic mice exhibited a significant upregulation of NLRP3, which were decreased by additional loss of GCN5L1 in the kidney compared with diabetic WT mice (Figure 5(c)). The same tendency was confirmed by western blotting analysis in the kidney (Figures 5(d) and 5(g)). Moreover, the renal fibrosis in diabetic mice was characterized by the loss of E-cadherin and the excess of *α*-SMA, which was almost completely reversed by GCN5L1 knockdown (Figures 5(c)–5(f)). Taken together, these results support that suppression of GCN5L1 protects the kidney from hyperglycemia-induced EMT and inflammation via the MnSOD/ROS pathway.

### 3.6. STZ Treatment Increases GCN5L1 Expression by Reducing Its Ubiquitination

**Figure 6:**
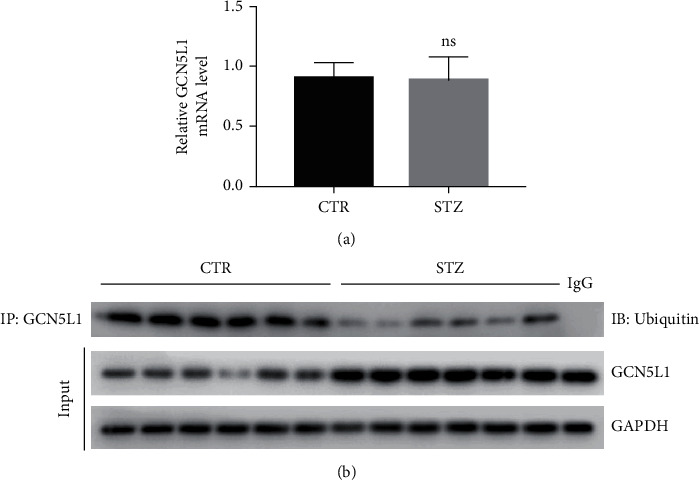
STZ treatment increases GCN5L1 expression by reducing its ubiquitination. (a) RNA levels of GCN5L1 were detected by RT-qPCR in the mouse kidneys. (b) The ubiquitination level of GCN5L1 was detected by immunoprecipitation assay. *n* = 6; ns: not significant.

In order to study the mechanism of increased GCN5L1 expression with STZ treatment, we first detected the mRNA expression of GCN5L1 under STZ treatment. According to the result of RT-qPCR, we found that this mRNA expression had no significant changes between the control and STZ groups (Figure 6(a)). Thus, we hypothesized that STZ treatment may increase GCN5L1 expression through a posttranslational mechanism. Ubiquitination is crucial for physiological processes which are controlled by ubiquitin and deubiquitinating enzymes [24, 25]. One of the important cellular functions of the ubiquitin-proteasome system (UPS) is to selectively degrade damaged or abnormal proteins [26]. UPS has an important role in diabetic nephropathy [27]. Mitochondria possess about 1,000-1,500 proteins with multiple functions, and thus, the quality control of the mitochondrial proteome handled by UPS is very important for mitochondrial function. Thus, dysfunction of UPS may contribute to mitochondrial dysfunction and DN. Under these backgrounds, we used immunoprecipitation assay to detect the ubiquitination level of GCN5L1. The results showed that the ubiquitination level of GCN5L1 was decreased under the STZ treatment, which induced the GCN5L1 overexpression in protein level (Figure 6(b)). According to all of these results, we found that GCN5L1 overexpression was controlled by impaired ubiquitination.

## 4. Discussion

Accumulating evidences have identified that hyperglycemia-induced oxidative stress is of particular interest in the development and progression of DKD [2, 28], but its exact mechanisms involved remain largely unknown. A new sight has been established that the high expression of GCN5L1 contributes to oxidative stress-induced renal tubulointerstitial injury in DKD by mediating MnSOD hyperacetylation and inactivating its enzymatic detoxification activity. Collectively, we identified that GCN5L1 could participate in cellular redox homeostasis maintenance in DKD.

GCN5L1, given its sequence homology to the nuclear acetyltransferase Gcn5 and its mitochondrial enrichment, has recently been recognized as an indispensable element in the machinery of mitochondrial acetyltransferases and has been proved to counter the activity of SIRT3, a mitochondrial deacetylase protein [11]. The pathogenic role of GCN5L1 has been reported in the past few years [29]. Study conducted in mouse embryonic fibroblasts illustrated that knockout of GCN5L1 has the effects on inducing mitochondrial biogenesis and mitophagy via the activation of PGC-1*α* and TFEB [12]. Upregulation of cardiac GCN5L1 in response to high-fat diet participates to the fatty acid oxidation rate by increasing the level of acetylation and the activity of FAO enzyme [13]. Furthermore, another study reported that depletion of GCN5L1 mediated ERK activation and blunted hepatic gluconeogenesis [30]. In renal disease, we previously demonstrated that GCN5L1 could upregulate both the acetylation and activity of several mitochondrial fatty acid oxidation enzymes, thus regulating fatty acid oxidation in the kidney [18]. Nevertheless, the role of GCN5L1 in the initiation and progression of DKD is still unclear. In the present study, we concentrated on exploring the alteration of GCN5L1 in DKD and investigating the effect of downregulation of GCN5L1 on oxidative stress-induced renal tubulointerstitial injury in DKD. First, we found that GCN5L1 expression was significantly increased not only in the kidney tissues of DKD patients and mouse models but also in renal TECs treated with high glucose. Second, the renal tubulointerstitial injury was alleviated in STZ-DKD mice treated with sh-GCN5L1 AAV. Furthermore, the genetic ablation of GCN5L1 in TECs ameliorated oxidative stress-induced EMT and inflammation both in vivo and in vitro. Taken together, the results above suggest that GCN5L1 could affect oxidative stress-induced renal damage under hyperglycemic conditions and targeting GCN5L1 could be an attractive therapeutic target of DKD.

As one of the most energy-demanding organs, the kidney requires a great deal of mitochondria to produce sufficient adenosine triphosphate (ATP) through oxidative metabolism; at the same time, reactive oxygen species (ROS) were also generated as a by-product. It has been reported that hyperglycemia could induce excessive production of ROS, thereby leading to oxidative stress [31]. There are numerous evidences showing that enhanced oxidative stress (OS) is an important feature in CKD patients from early to late stage [32, 33]. Excessive OS is caused by the dysfunction of the balance between antioxidant defense mechanisms and oxidant products [34]. Therefore, the sources of excessive OS can be divided into two main parts. One is excessive OS produced like mitochondria dysfunction, disorder of metabolic networks, and impairment of mitophagy [35]. The other is the defect of the antioxidant system including the decrease of small-molecule antioxidants such as vitamins C and E and the tripeptide glutathione (GSH), dysfunction of enzymes whose specific role is the neutralization of ROS like superoxide dismutase (SOD), catalase, and GSH peroxidases (GPx) [36]. The mitochondrial antioxidant enzyme system is the major defense to eliminate superoxide free radicals, which is related to the pathophysiology of DKD.

As the key antioxidant enzyme in mitochondria, MnSOD could scavenge aberrant ROS to protect cells against oxidative stress. Several posttranslational modulation (PTM) patterns of MnSOD have been reported, such as nitration [37], acetylation [38], and ubiquitination [39], among which acetylation of MnSOD is one of the main drivers of its enzyme activity which has attracted widespread attention and been reported in various kinds of diseases, such as breast cancer [40] and cardiac diseases [41]. Current studies have revealed that elevation of lysine acetylation decreases MnSOD enzymatic activity, which is in accordance with our present study. The upstream mechanism of regulating MnSOD lysine acetylation is mostly concentrated on the mitochondrial deacetylase enzyme SIRT3, which deacetylated MnSOD on different reversible acetylated lysine residues. He and his colleagues have found that SIRT3-mediated deacetylation of lysine 68 regulates stem cell reprogramming in breast cancer [40]. Other researches have also reported that SIRT3 could also regulate the acetylation level of MnSOD lysine 53, 89, and 122 [10, 42]. In addition, Thapa and his colleagues reported that GCN5L1 is acetylating MnSOD at K122 in cardiomyocyte [43]. According to the published paper, acetylation of MnSOD K68 lysine residue can transform from a known homotetramer complex to a monomeric form. These monomers function as a peroxidase, distinct from the established MnSOD superoxide dismutase activity. Therefore, K68 lysine residue acetylation decreased the enzyme activity. Moreover, acetylation of other sites like K122 had no this effect [38]. According to all of these papers, we focused on K68 site and hypothesized that this site acetylation can lower the activity of MnSOD. According to our results, we shed new light on the mitochondrial acetyltransferase GCN5L1 which could acetylate MnSOD K68 lysine residues, thereby diminishing MnSOD enzymatic detoxification activity. Furthermore, enhanced abundance of MnSOD^K68^ acetylation and reduced MnSOD activity were displayed in the DKD mouse kidneys and hyperglycemic TECs. Moreover, silencing GCN5L1 declined MnSOD^K68^ acetylation state but improved its ability to scavenge ROS to prevent oxidative stress and cellular damage in DKD. Moreover, we found that GCN5L1 overexpression under STZ treatment was induced by impaired ubiquitination. The exact molecules controlled this process and their roles in DN should be studied further. In general, we have discovered the acetyltransferase GCN5L1 could acetylate MnSOD, providing a new mechanism to improve renal injury in DKD.

## 5. Conclusions

In summary, our present study demonstrated that GCN5L1 mediates MnSOD acetylation on lysine 68 to regulate its detoxification activity, thereby affecting oxidative stress-induced renal tubulointerstitial injury in DKD. Hence, GCN5L1 might be a key regulator of renal oxidative stress and function as a potential therapeutic target in DKD.

## 6. Limitations of the Study

First, although we used adenovirus-associated virus to knock down GCN5L1 in the mouse kidneys to explore the role of GCN5L1 in DKD, it would be perfect to apply conditional TECs GCN5L1 knockout mice. Second, we explored that GCN5L1 abundance under STZ treatment was dependent on a posttranslational mechanism, but which molecules controlled this process and their roles in DN were unclear.

## Data Availability

The data supporting the findings of this study could be obtained from the corresponding author.

## Supplementary Materials

Supplementary Materials Supplementary Table S1: the blood glucose data of mice. Supplementary Figure S1: the actual blood glucose data of mice. Supplementary Figure S2: NAC impairs the NLRP3 activation and EMT induced by GCN5L1 overexpression. Three times of independent experiments were performed for detecting the effect of NAC on protein expression. Supplementary Figure S3: NAC has no significant effect on MnSOD activity and acetylation. (a) MnSOD activity was assessed in TECs treated with NAC. (b) Western blotting analysis of the protein levels of Ac-MnSOD K68 in TECs treated with NAC.

## References

[1] Martínez-Castelao A., Navarro-González J., Górriz J., de Alvaro F. (2015). The concept and the epidemiology of diabetic nephropathy have changed in recent years. *Journal of Clinical Medicine*.

[2] Jha J. C., Banal C., Chow B. S. M., Cooper M. E., Jandeleit-Dahm K. (2016). Diabetes and kidney disease: role of oxidative stress. *Antioxidants & Redox Signaling*.

[3] Ahmad J. (2015). Management of diabetic nephropathy: recent progress and future perspective. *Diabetes & Metabolic Syndrome: Clinical Research & Reviews*.

[4] Bekele B. B. (2019). The prevalence of macro and microvascular complications of DM among patients in Ethiopia 1990-2017: systematic review. *Diabetes & Metabolic Syndrome: Clinical Research & Reviews*.

[5] Xue R., Gui D., Zheng L., Zhai R., Wang F., Wang N. (2017). Mechanistic insight and management of diabetic nephropathy: recent progress and future perspective. *Journal of Diabetes Research*.

[6] Hirakawa Y., Nangaku M. (2018). Targeting oxidative stress in diabetic kidney disease: a novel drug in an old pathway. *Kidney International*.

[7] Wang Y., Branicky R., Noe A., Hekimi S. (2018). Superoxide dismutases: dual roles in controlling ROS damage and regulating ROS signaling. *The Journal of Cell Biology*.

[8] Fukai T., Ushio-Fukai M. (2011). Superoxide dismutases: role in redox signaling, vascular function, and diseases. *Antioxidants & Redox Signaling*.

[9] Ozden O., Park S.-H., Kim H.-S. (2011). Acetylation of MnSOD directs enzymatic activity responding to cellular nutrient status or oxidative stress. *Aging*.

[10] Tao R., Coleman M. C., Pennington J. D. (2010). Sirt3-mediated deacetylation of evolutionarily conserved lysine 122 regulates MnSOD activity in response to stress. *Molecular Cell*.

[11] Scott I., Webster B. R., Li J. H., Sack M. N. (2012). Identification of a molecular component of the mitochondrial acetyltransferase programme: a novel role for GCN5L1. *The Biochemical Journal*.

[12] Scott I., Webster B. R., Chan C. K., Okonkwo J. U., Han K., Sack M. N. (2014). GCN5-like protein 1 (GCN5L1) controls mitochondrial content through coordinated regulation of mitochondrial biogenesis and mitophagy. *Journal of Biological Chemistry*.

[13] Thapa D., Zhang M., Manning J. R. (2017). Acetylation of mitochondrial proteins by GCN5L1 promotes enhanced fatty acid oxidation in the heart. *American Journal of Physiology Heart and Circulatory Physiology*.

[14] Webster B. R., Scott I., Han K. (2013). Restricted mitochondrial protein acetylation initiates mitochondrial autophagy. *Journal of Cell Science*.

[15] Cheng R.-X., Feng Y., Liu D. (2019). The role of Nav1.7 and methylglyoxal-mediated activation of TRPA1 in itch and hypoalgesia in a murine model of type 1 diabetes. *Theranostics*.

[16] Liu Y., Yang Z., Kong D., Zhang Y., Yu W., Zha W. (2019). Metformin ameliorates testicular damage in male mice with streptozotocin- induced type 1 diabetes through the PK2/PKR pathway. *Oxidative Medicine and Cellular Longevity*.

[17] Yang Y.-L., Xue M., Jia Y.-J. (2020). Long noncoding RNA NEAT1 is involved in the protective effect of Klotho on renal tubular epithelial cells in diabetic kidney disease through the ERK1/2 signaling pathway. *Experimental & Molecular Medicine*.

[18] Lv T., Hu Y., Ma Y., Zhen J., Xin W., Wan Q. (2019). GCN5L1 controls renal lipotoxicity through regulating acetylation of fatty acid oxidation enzymes. *Journal of Physiology and Biochemistry*.

[19] Ma Y., Zhu S., Lv T. (2020). SQSTM1/p62 controls mtDNA expression and participates in mitochondrial energetic adaption via MRPL12. *iScience*.

[20] Tuttle K. R., Bakris G. L., Bilous R. W. (2014). Diabetic kidney disease: a report from an ADA Consensus Conference. *Diabetes Care*.

[21] Ding T., Wang S., Zhang X. (2018). Kidney protection effects of dihydroquercetin on diabetic nephropathy through suppressing ROS and NLRP3 inflammasome. *Phytomedicine*.

[22] Minutoli L., Puzzolo D., Rinaldi M. (2016). ROS-mediated NLRP3 inflammasome activation in brain, heart, kidney, and testis ischemia/reperfusion injury. *Oxidative Medicine and Cellular Longevity*.

[23] Rhyu D. Y., Yang Y., Ha H. (2005). Role of reactive oxygen species in TGF-*β*1-induced mitogen-activated protein kinase activation and epithelial-mesenchymal transition in renal tubular epithelial cells. *Journal of the American Society of Nephrology*.

[24] Popovic D., Vucic D., Dikic I. (2014). Ubiquitination in disease pathogenesis and treatment. *Nature Medicine*.

[25] Swatek K. N., Komander D. (2016). Ubiquitin modifications. *Cell Research*.

[26] Cybulsky A. V. (2013). The intersecting roles of endoplasmic reticulum stress, ubiquitin-proteasome system, and autophagy in the pathogenesis of proteinuric kidney disease. *Kidney International*.

[27] Goru S. K., Kadakol A., Gaikwad A. B. (2017). Hidden targets of ubiquitin proteasome system: to prevent diabetic nephropathy. *Pharmacological Research*.

[28] Stanton R. C. (2011). Oxidative stress and diabetic kidney disease. *Current Diabetes Reports*.

[29] Scott I., Wang L., Wu K., Thapa D., Sack M. N. (2018). GCN5L1/BLOS1 links acetylation, organelle remodeling, and metabolism. *Trends in Cell Biology*.

[30] Wang L., Scott I., Zhu L. (2017). GCN5L1 modulates cross-talk between mitochondria and cell signaling to regulate FoxO1 stability and gluconeogenesis. *Nature Communications*.

[31] Yuan T., Yang T., Chen H. (2019). New insights into oxidative stress and inflammation during diabetes mellitus- accelerated atherosclerosis. *Redox Biology*.

[32] Liakopoulos V., Roumeliotis S., Gorny X., Dounousi E., Mertens P. R. (2017). Oxidative stress in hemodialysis patients: a review of the literature. *Oxidative Medicine and Cellular Longevity*.

[33] Duni A., Liakopoulos V., Roumeliotis S., Peschos D., Dounousi E. (2019). Oxidative stress in the pathogenesis and evolution of chronic kidney disease: untangling Ariadne’s thread. *International Journal of Molecular Sciences*.

[34] Locatelli F., Canaud B., Eckardt K. U., Stenvinkel P., Wanner C., Zoccali C. (2003). Oxidative stress in end-stage renal disease: an emerging threat to patient outcome. *Nephrology Dialysis Transplantation*.

[35] Filomeni G., De Zio D., Cecconi F. (2015). Oxidative stress and autophagy: the clash between damage and metabolic needs. *Cell Death & Differentiation*.

[36] Lewerenz J., Hewett S. J., Huang Y. (2013). The cystine/glutamate antiporter system x(c)(-) in health and disease: from molecular mechanisms to novel therapeutic opportunities. *Antioxidants & Redox Signaling*.

[37] Redondo-Horcajo M., Romero N., Martínez-Acedo P. (2010). Cyclosporine A-induced nitration of tyrosine 34 MnSOD in endothelial cells: role of mitochondrial superoxide. *Cardiovascular Research*.

[38] Zhu Y., Zou X., Dean A. E. (2019). Lysine 68 acetylation directs MnSOD as a tetrameric detoxification complex versus a monomeric tumor promoter. *Nature Communications*.

[39] Takabe W., Li R., Ai L., Yu F., Berliner J. A., Hsiai T. K. (2010). Oxidized low-density lipoprotein-activated c-Jun NH2-terminal kinase regulates manganese superoxide dismutase ubiquitination. *Arteriosclerosis, Thrombosis, and Vascular Biology*.

[40] He C., Danes J. M., Hart P. C. (2019). SOD2 acetylation on lysine 68 promotes stem cell reprogramming in breast cancer. *Proceedings of the National Academy of Sciences*.

[41] Dikalova A. E., Itani H. A., Nazarewicz R. R. (2017). Sirt3 impairment and SOD2 hyperacetylation in vascular oxidative stress and hypertension. *Circulation Research*.

[42] Qiu X., Brown K., Hirschey M. D., Verdin E., Chen D. (2010). Calorie restriction reduces oxidative stress by SIRT3-mediated SOD2 activation. *Cell Metabolism*.

[43] Thapa D., Manning J. R., Stoner M. W., Zhang M., Xie B., Scott I. (2020). Cardiomyocyte-specific deletion of GCN5L1 in mice restricts mitochondrial protein hyperacetylation in response to a high fat diet. *Scientific Reports*.

