# Supplementary material for: General Control of Amino Acid Synthesis 5-Like 1-Mediated Acetylation of Manganese Superoxide Dismutase Regulates Oxidative Stress in Diabetic Kidney Disease
Source: Oxid Med Cell Longev. 2021 Feb 17;2021:6691226. doi: 10.1155/2021/6691226 (PMC7906818; doi:10.1155/2021/6691226)
Supplement: Supplementary Materials — Supplementary Table S1: the blood glucose data of mice. Supplementary Figure S1: the actual blood glucose data of mice. Supplementary Figure S2: NAC impairs the NLRP3 activation and EMT induced by GCN5L1 overexpression. Three times of independent experiments were performed for detecting the effect of NAC on protein expression. Supplementary Figure S3: NAC has no significant effect on MnSOD activity and acetylation. (a) MnSOD activity was assessed in TECs treated with NAC. (b) Western blotting analysis of the protein levels of Ac-MnSOD K68 in TECs treated with NAC. [file 6691226.f1.docx]

**Supplementary Table S1: The blood glucose data of mice.**

| Ear Tag Number | 101 | 102 | 103 | 104 | 105 | 106 |  |  |  |
| --- | --- | --- | --- | --- | --- | --- | --- | --- | --- |
| non-DN group Blood glucose (mmol/l) | 6.1 | 5.6 | 4.6 | 7.2 | 6.4 | 7.8 |  |  |  |
|  | 7.2 | 8.1 | 7.3 | 4.6 | 5.8 | 7.4 |  |  |  |
|  | 5.4 | 7.6 | 8.1 | 5.7 | 7.5 | 6.4 |  |  |  |
| Ear Tag Number | 111 | 112 | 113 | 114 | 115 | 116 | 117 | 118 | 119 |
| DN group Blood glucose (mmol/l) | 25.3 | 25.6 | 26.3 | 23.8 | 24.5 | 22.6 | 11.2 | 9.6 | 10.2 |
|  | 20.9 | 22.4 | 24.6 | 22.7 | 23.8 | 26.4 | 13.6 | 8.2 | 11.9 |
|  | 21.8 | 24.2 | 21.6 | 25.2 | 26.2 | 28.2 | 9.8 | 6.8 | 8.5 |
| Ear Tag Number | 121 | 122 | 123 | 124 | 125 | 126 | 127 | 128 | 129 |
| AAV-vector DN group Blood glucose (mmol/l) | 20.2 | 20.4 | 21.5 | 26.4 | 29.8 | 23.2 | 26.6 | 28 | 11.8 |
|  | 22.6 | 21.5 | 19.8 | 27.6 | 30.2 | 20.8 | 24.2 | 27.8 | 12.6 |
|  | 26.4 | 24.3 | 20.4 | 28.9 | 28.6 | 24.2 | 22.1 | 26.9 | 13.2 |
| Ear Tag Number | 131 | 132 | 133 | 134 | 135 | 136 | 137 | 138 | 139 |
| AAV-GCN5L1 DN group Blood glucose (mmol/l) | 24.2 | 20.2 | 22.1 | 25.2 | 24.6 | 23.4 | 24.2 | 20.6 | 9.8 |
|  | 26.8 | 21.5 | 23.4 | 26.3 | 26.4 | 25.6 | 22.2 | 21.6 | 11.4 |
|  | 25.3 | 24.1 | 20.8 | 24.2 | 20.8 | 21.4 | 20.1 | 24.2 | 10.2 |


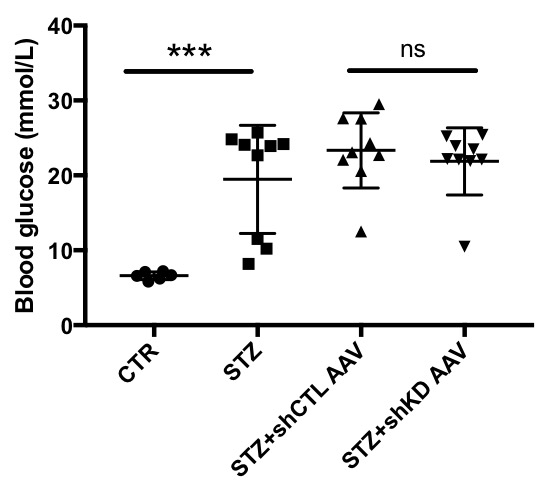


**Supplementary figureS1:** The actual blood glucose data of mice.


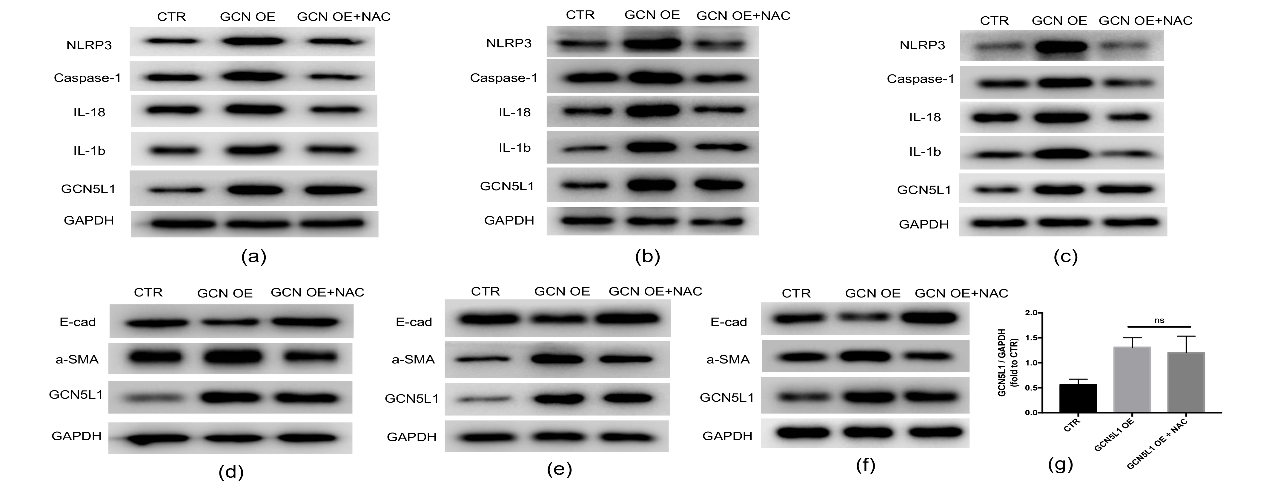


**Supplementary figureS2**: NAC impairs the NLRP3 activation and EMT induced by GCN5L1 overexpression. Three times of independent experiments were performed for detecting the effect of NAC on proteins expression.


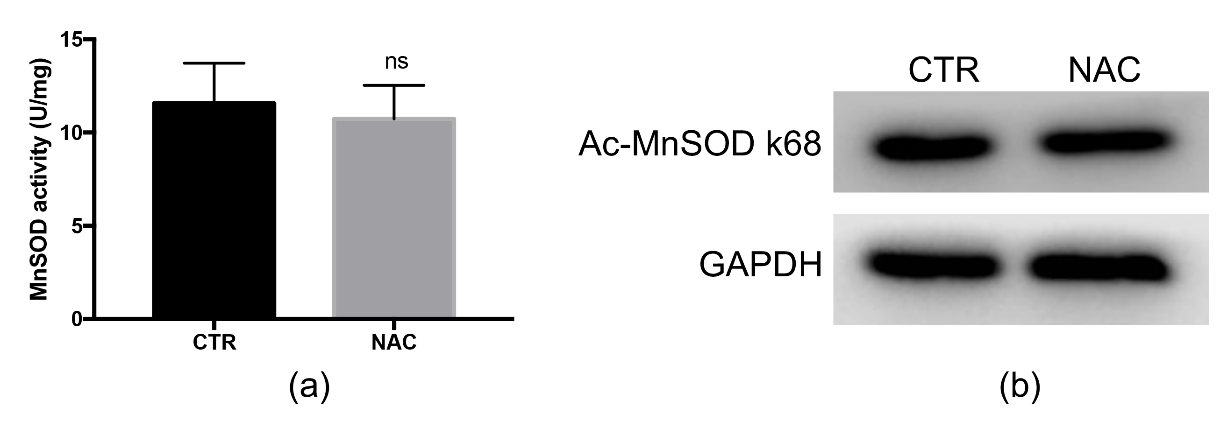


**Supplementary figureS3**: NAC has no significant effect on MnSOD activity and acetylation. (a) MnSOD activity was assessed in TECs treated with NAC. (b) Western blotting analysis of the protein levels of Ac-MnSOD K68 in TECs treated with NAC.
